# Safety and tolerance of lymph node biopsies from chronic HIV-1 volunteers in rural Tanzania

**DOI:** 10.1186/s13104-019-4600-x

**Published:** 2019-09-06

**Authors:** Catherine Gerald Mkindi, Elias Antony Marandu, Ngisi Masawa, Farida Bani, Amina Nyuri, Theonestina Byakuzana, Thomas Klimkait, Song Ding, Giuseppe Pantaleo, Manuel Battegay, Nina Orlova-Fink, Maja Weisser-Rohacek, Claudia Daubenberger

**Affiliations:** 1 Department of Medical Parasitology and Infection Biology, Clinical Immunology, Swiss Tropical and Public Health Institute, Socinstr. 57, 4002 Basel, Switzerland; 2 University of Basel, Basel, Switzerland; 3 Ifakara Health Institute, Bagamoyo, Tanzania; 4 Division of Infectious Diseases and Hospital Epidemiology, University Hospital Basel, Basel, Switzerland; 5 St Francis Referral Hospital, Ifakara, Tanzania; 6 EuroVacc Foundation, Amsterdam, The Netherlands; 7 Service of Immunology and Allergy, Lausanne University Hospital, Lausanne, Switzerland; 8 Swiss Vaccine Research Institute, Lausanne, Switzerland

**Keywords:** Lymph node biopsy, KIULARCO cohort, Tanzania, HIV-1, Chronic, Elite controller

## Abstract

**Objective:**

HIV-1 rapidly establishes a persistent infection that can be contained under life-long antiretroviral therapy (ART) but not cured. One major viral reservoir is the peripheral lymph node (LN) follicles. Studying the impact of novel HIV-1 treatment and vaccination approaches on cells residing in germinal centers is essential for rapid progress towards HIV-1 prevention and cure.

**Results:**

We enrolled 9 asymptomatic adult volunteers with a newly diagnosed HIV-1 infection and CD4 T cell counts ≥ 350/ml. The patients underwent venous blood collection and inguinal lymph node excision surgery in parallel. Mononuclear cells were extracted from blood and tissues simultaneously. Participants were followed up regularly for 2 weeks until complete healing of the surgical wounds. All participants completed the lymph node excision surgery without clinical complications. Among the 9 volunteers, one elite controller was identified. The number of mononuclear cells recovered from lymph nodes ranged from 68 to 206 million and correlated positively with lymph node size. This is the first study to show that lymph node biopsy is a safe procedure and can be undertaken with local experts in rural settings. It provides a foundation for detailed immune response investigations during future clinical trials.

## Introduction

Human immunodeficiency virus type 1 (HIV-1) has become one of the most serious global health challenges, despite intensive research over the last 30 years. In 2017, of the estimated 36.9 million people living with HIV (PLWHIV) worldwide, 19.6 million resided in eastern and southern Africa [1]. The UNAIDS goals of 90-90-90 by 2020 (90% diagnosed, 90% treated, 90% virally suppressed), are not yet met in Tanzania: 66% of those diagnosed with HIV-1 are on ART and 48% of these have suppressed viremia, while no numbers are published for the first 90 [1].

Most current vaccines provide protection by generating antibodies that neutralize pathogen entry or spread [2]. Numerous efforts to elicit broadly neutralizing antibodies (bnAbs) against HIV in experimental animals and humans have been unsuccessful [3]. However, many HIV-1 infected patients naturally develop bnAbs that can effectively neutralize a broad range of HIV-1 variants and suppress viremia [4]. The development of such potent antibodies usually takes several years and is poorly understood as selection takes place in the germinal centers (GC) located in secondary lymphoid organs, where B cells closely interact with T-follicular helper cells (Tfh), T follicular regulatory (Tfr) cells, macrophages and follicular dendritic cells [5, 6]. Studying these cell populations and their interactions is of a great importance but has so far been very limited, since access to those tissues is challenging. In humans, several markers for Tfh circulating in peripheral blood have been established, including ICOS, PD1 and CXCR5 expression [7], enabling the investigation of vaccine-induced responses against vaccination in peripheral blood [8, 9]. However, the functional and biological relationship between circulating Tfh and cells residing in secondary lymphoid organs is unclear and requires further investigation [10].

The Kilombero and Ulanga Antiretroviral Cohort (KIULARCO) is a single-site, open and ongoing prospective cohort of HIV-1 patients established in 2005 at the Chronic Diseases Clinic of Ifakara (CDCI), located at the Saint Francis Referral Hospital (SFRH) in Ifakara, Tanzania [11]. One of the objectives of KIULARCO is to provide a platform for clinical studies on improving HIV-1 care and treatment [12]. Here, as a proof of concept, we performed a lymph node excision study from asymptomatic, chronically HIV-1-infected volunteers in this rural setting in Tanzania.

## Main text

### Results

Nine ART-naïve, HIV-1-positive volunteers were enrolled into the study between June and August 2018. Volunteers reported that they came to SFRH for reasons other than acute sickness or suspicion of HIV infection (Table 1). At enrolment, all participants were clinically healthy, with no fever, chills or headache recorded. The female-to-male ratio was 3:1 and the average age of the volunteers was 34 years (range 23–55 years). CD4 T-cell counts were on average 698 cells/ml (range 434–1302) and the HIV-1 viral loads, assessed at the time of diagnosis, measured on average 35,500 copies/ml (range < 50 to 133,200).

**Table 1:** Demographic characteristics and clinical data of the volunteers

	Volunteer ID								
	V1	V2	V3	V4	V5	V6	V7	V8	V9
Age	40	55	42	29	23	23	37	26	30
Gender	F	F	F	F	F	M	F	M	M
BMI	18.7	22.2	28.4	24.9	21.3	23	31.6	18.8	29.7
Marital status	Divorced	Divorced	Divorced	Single	Single	Married	Married	Single	Single
CD4 count (cells/ml)	538	662	1302	846	679	594	434	665	566
HIV-1 RNA (copies/ml)	1670	68,300	< 50	8150	725	113,200	72,200	53,900	1210
Hb (g/l)	13.9	12.0	12.1	12.7	10.2	14.1	13.8	15.0	13.7
Syphilis	Negative	Negative	Negative	Negative	Negative	Negative	Negative	Negative	Negative
Hepatitis B	Negative	Negative	Negative	Negative	Negative	Negative	Negative	Negative	Negative
Clinical presentation	Healthy	Healthy	Healthy	Healthy	Healthy	Healthy	Healthy	Healthy	Healthy
Reason for HIV-1 testing	Ear injury	History of herpes zoster lesions	Chronic body malaise	Marital requirement	Employer request	Wife’s antenatal clinic visit	Referred	Voluntary Testing	Voluntary Testing

For all participants the duration of infection is unknown, but given their asymptomatic conditions, relatively moderate viral loads and healthy CD4 T-cell counts, it is safe to assume that the participants have a chronic HIV-1 infection.

**Fig. 1:**
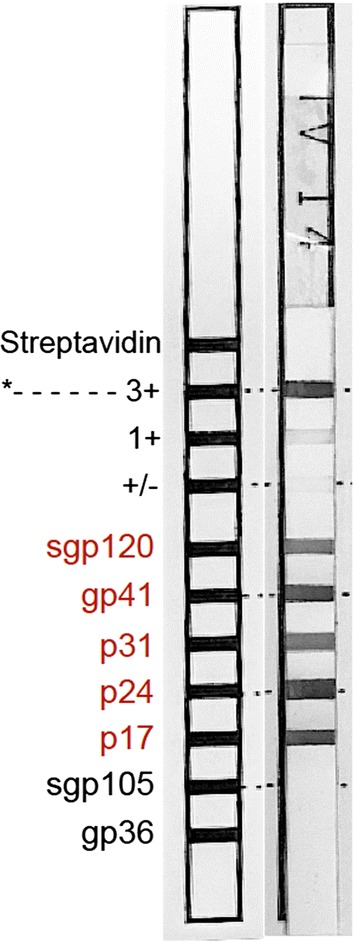
Detection of HIV-1 specific antibodies in participant V3 by a line immunoassay (HIV-1 detection bands indicated in red, HIV-2 in black)

Interestingly, one of the volunteers (V3), who had a positive diagnosis for HIV-1 with two different RDTs, showed undetectable HIV-1 viral load (< 50 copies/ml) and high CD4 T-cell count (1302 cells/ml), the highest in this cohort. HIV-1, but not HIV-2, infection for this volunteer was confirmed by additional testing with a line immunoassay (Fig. 1) suggesting a rare elite controller phenotype.

**Fig. 2:**
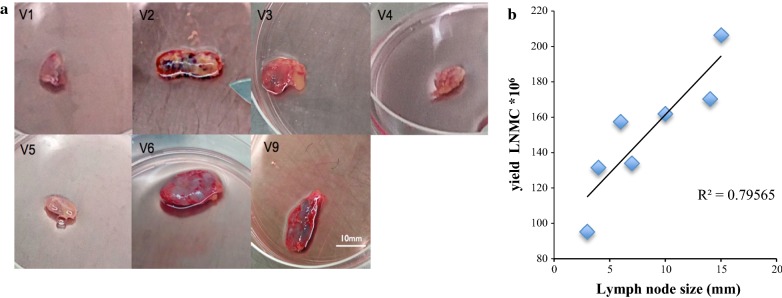
**a** Pictures of lymph node biopsies taken from volunteers. **b** Positive correlation between LN size and cell yield

Lymph node biopsies were successfully conducted from all 9 volunteers and the sizes of extracted nodes ranged from 3 to 15 mm (Fig. 2a). After extraction, LMNC were immediately isolated in the closely located laboratory. Viability of LMNC ranged from 97 to 99% and the count of recovered LMNC ranged from 68 to 206 million. The LNMC yield correlated positively with the lymph node size (Fig. 2b), supporting strongly that healthy secondary lymphoid organs were extracted. Cell samples were aliquoted and stored within an hour after surgical excision. Except for two volunteers, who developed self-resolving hyperaemia surrounding the incision site, none of the volunteers experienced any complications in relation to the lymph node biopsy. The incisons healed as expected and all volunteers were discharged from attending regular hospital visits by day 14 the latest. Volunteers started HIV-1 treatment on the day of enrolment in KIULARCO, as recommended by the national treatment guidelines of Tanzania.

### Discussion

Biopsies of inguinal lymph nodes together with a paired venous blood sample were collected from nine HIV-1 infected patients in rural settings in Tanzania. To our knowledge, this is the first study of this kind in Tanzania. We demonstrate that this procedure is safe and well-tolerated with minimal adverse events that can resolve within 14 days after surgery. The short recruitment period of volunteers into the study demonstrates that the KIULARCO cohort serves as an elegant platform for clinical HIV research. HIV-1 is not curable using the currently available treatment approaches, resulting in a growing number of PLWHIV who need to adhere to life-long ART treatment [13]. One of the common compartments for viral persistence is Tfh cells, making them an important target for experimental approaches to a cure for HIV [14].

A recent report by D´Souza provided an excellent overview of the value of lymph node biopsies in the context of experimental HIV-1 vaccine trials [15]. Lymph node biopsies have been shown to be safe in HIV-1-positive volunteers, with up to 4 lymph nodes able to be consecutively removed from participants during a HIV-1 vaccine study in Thailand [16]. The bulk of information generated about GC development and responses has been derived from studies on secondary lymphoid tissues in mice. It is assumed that heterogeneity of GC Tfh cells impacts antibody isotypes produced by plasma blasts and long-lived memory B cells [17]. In humans, GC Tfh are a functionally and phenotypically heterogeneous population based on the expression of PD1, CXCR3, CCR6, CCR7 and ICOS markers [18]. People living in SSA are exposed to co-infections such as malaria and helminths, which potentially impact GC Tfh cell function and cytokine secretion [19]. Studying the environmental drivers of the functional heterogeneity of GC Tfh in a rural African population will provide essential information on HIV pathogenesis, treatment as well as routine and experimental vaccine monitoring in a population highly affected by the HIV epidemic [20].

### Conclusions

Inguinal lymph node excisions can be safely performed by local experts in a rural sub-Saharan African setting, so long as precautions such as infection prevention are in place and followed carefully. Thus, the procedure is a feasible practice in the framework of monitoring novel intervention studies in HIV-1 clinical research. The KIULARCO cohort provides a valuable platform for clinical research, supporting the evaluation of novel interventions in a population that is highly affected by HIV-1 but rather neglected in relation of testing novel interventions.

### Methods

#### Study site

This study was conducted in the rural communities of Morogoro region in Tanzania, involving two districts: Kilombero and Ulanga. The Chronic Diseases Clinic of Ifakara (CDCI) located at the St Francis Referral Hospital (SFRH) runs an antiretroviral cohort (Kilombero and Ulanga Antiretroviral Cohort, KIULARCO). KIULARCO includes all HIV-1-positive patients enrolled in care. SFRH has in- and outpatient services and specialized clinics including theatres with facilities needed for patient recruitment and lymph node extraction. The Ifakara Health Institute runs an advanced laboratory infrastructure, supporting immunological and virological sample processing and long-term storage.

#### Volunteer recruitment and diagnostics

All patients seen for any health condition at SFRH are offered an HIV-1 test following the national strategic plan [11]. In 2014, SFRH adopted the WHO strategy for universal HIV-1 testing for all visitors, regardless of health condition. Routine testing for HIV-1 is done by a rapid diagnostic test (RDT) (SD Bioline), confirmed by a secondary RDT (Unigold) where necessary. HIV-1 viral RNA plasma loads are quantified by using GenXpert with a detection limit of 50 copies/ml. For confirmation of HIV infection and differentiation between HIV-1 and HIV-2, the line immunoassay, INNO-LIA HIV I/II Score (Fujirebio, Ghent, Belgium) (Inno-Lia), had been used in one volunteer (V3), whose viral load was below the limit of detection by GenXpert.

HIV-1 positive volunteers were eligible for enrolment dependent on meeting the following criteria: between 18 and 55 years of age, newly diagnosed (ART-naïve), CD4 T-cell count above 350 cells/ml, no active co-morbidities, negative for hepatitis B (HBsAg), Syphilis and malaria. Volunteers were informed about study procedures and consented to paired blood and lymph node removal. All volunteers were recruited into the KIULARCO on the same day, after consenting, and provided with ART treatment.

#### Blood sample collection and PBMC isolation

Whole blood was collected from all participants on the day of the LN biopsies. Plasma and serum samples were prepared following established procedures [12] and stored at − 80 °C. PBMCs were isolated by density gradient centrifugation using Ficoll-Hypaque and stored in Fetal calf serum (FCS) with 10% DMSO in liquid nitrogen until further analysis.

#### Lymph node extraction and cell isolation

A surgical procedure to excise a single inguinal LN from each volunteer was performed aseptically at the SFRH theatre following local anaesthesia, 2% lidocaine 5 ml. An incision of 1–2 cm in length was made at the right or left inguinal area. LNs were immediately preserved in R10 medium (RPMI with 1% Penicillin/Streptomycin and 10% FCS) for transport and processing. The incisions were sutured and patients were prescribed analgesics before being discharged. Patients were asked to come for three visits within 14 days of surgery for postoperative wound care. Lymph node mononuclear cells (LNMC) were extracted in R10 medium by mechanical disruption of the tissue causing cell release, followed by filtering with a 100un filter. Cell counts and viability were determined by microscopy using 0.4% trypan blue solution staining.

## Limitations

The study was designed as a pilot study, therefore included a limited number of the participants.

## Data Availability

Not applicable.

## Abbreviations

ART: antiretroviral therapy
bnABs: broadly neutralizing antibodies
CDCI: Chronic Diseases Clinic of Ifakara
DMSO: dimethyl sulfoxide
FCS: fetal calf serum
GC: germinal center
HIV-1: human immunodeficiency virus type 1
KIULARCO: Kilombero and Ulanga Antiretroviral Cohort
LN: lymph node
LNMC: lymph node mononuclear cells
PLWHIV: people living with HIV
RDT: rapid diagnostic test
SFRH: Saint Francis Referral Hospital, Ifakara, Tanzania
SSA: sub**-**Saharan Africa
Tfh: follicular B helper T cells
Tfr: follicular regulatory T cells
UNAIDS: Joint United Nations Programme on HIV and AIDS
WHO: World Health Organization
